# EZH2 inhibition induces pyroptosis via RHA-mediated S100A9 overexpression in myelodysplastic syndromes

**DOI:** 10.1186/s40164-025-00600-3

**Published:** 2025-01-29

**Authors:** Qi Zhang, Yingwan Luo, Li Ye, Yuxia Wang, Lu Wang, Wenli Yang, Wei Lang, Shuanghong Zhu, Lingxu Jiang, Weimei Jin, Chen Mei, Xinping Zhou, Yanling Ren, Liya Ma, Gaixiang Xu, Bowatte Gedara Lakmal Vimukthi Bandara Bowattage, Hongyan Tong, Jie Sun

**Affiliations:** 1 Department of Hematology, the First Affiliated Hospital, Zhejiang University School of Medicine, Hangzhou, Zhejiang China; 2 Zhejiang Provincial Key Laboratory of Hematopoietic Malignancy, Zhejiang University , Hangzhou, Zhejiang China; 3 Zhejiang Provincial Clinical Research Center for Hematological Disorders, Hangzhou, China; 4 Zhejiang University Cancer Center, Hangzhou, Zhejiang China

**Keywords:** Myelodysplastic syndromes, Enhancer of zeste homologue 2, RNA helicase A, S100 calcium binding protein A9, Pyroptosis

## Abstract

Myelodysplastic Syndromes (MDS) represent a group of heterogeneous myeloid clonal diseases derived from aberrant hematopoietic stem/progenitor cells. Enhancer of zeste homolog 2 (EZH2) is an important regulator in gene expression through methyltransferase-dependent or methyltransferase-independent mechanisms. Herein, we found EZH2 inhibition led to MDS cell pyroptosis through RNA Helicase A (RHA) down-regulation induced overexpression of S100A9, a key regulator of inflammasome activation and pyroptosis. Moreover, EZH2 inhibitor reduced tumor burden and prolonged the survival of the mice transplanted with MDS cells. In summary, our results uncovered a novel pyroptosis pathway induced by EZH2 inhibition and provided a rationale for EZH2 inhibitor treatment in MDS.

**Supplementary Information:**

The online version contains supplementary material available at 10.1186/s40164-025-00600-3.

To the Editor,

Myelodysplastic Syndromes (MDS) are a group of myeloid disorders originating from hematopoietic stem/progenitor cells, characterized by peripheral cytopenia, dysplasia and high-risk transformation to acute myeloid leukemia (AML). Enhancer of zeste homolog 2 (EZH2) was initially described as a histone methyltransferase (HMT) that repressed gene expression by trimethylation of lysine 27 on histone H3. However, accumulating studies suggested EZH2 also acted as a transcriptional coactivator independent of the HMT activity and played crucial roles in cancers [1].

DZNep was reported to induce EZH2 protein degradation [2]. We found that treatment of DZNep inhibited the growth of MDS cells in vitro, reduced engraftment ability of MDS cell, and prolonged the overall survival of MDS cell transplanted mice (Fig. 1A-G, Fig. S1A). Interestingly, DZNep treatment led to about 35% of MDS cells exhibiting cell swelling with large bubbles appearing on the cellular membrane (Fig. 1H), a hallmark of cell pyroptosis mediated by pathogen-associated molecular patterns and damage-associated molecular patterns (DAMPs) [3, 4]. To confirm pyroptosis, we pretreated MDS cells with various cell death inhibitors before administering DZNep and found the caspase-1 inhibitor (VX765) restored cell viability (Fig. S1B). DZNep significantly increased NLRP3 expression (Fig. 1I), essential for inflammasome activation, indicating that DZNep treatment induces pyroptosis [5]. When activated, caspase-1 cleaves gasdermin D (GSDMD) into GSDMD-N, which promotes the release of IL-18 and eventually leads to pyroptosis [3]. Consistently, reduced EZH2 level with DZNep or its specific shRNA resulted in cell pyroptosis as evidenced by increased caspase-1 and GSDMD-N and IL-18 generation (Fig. 1J-K).

**Fig. 1:**
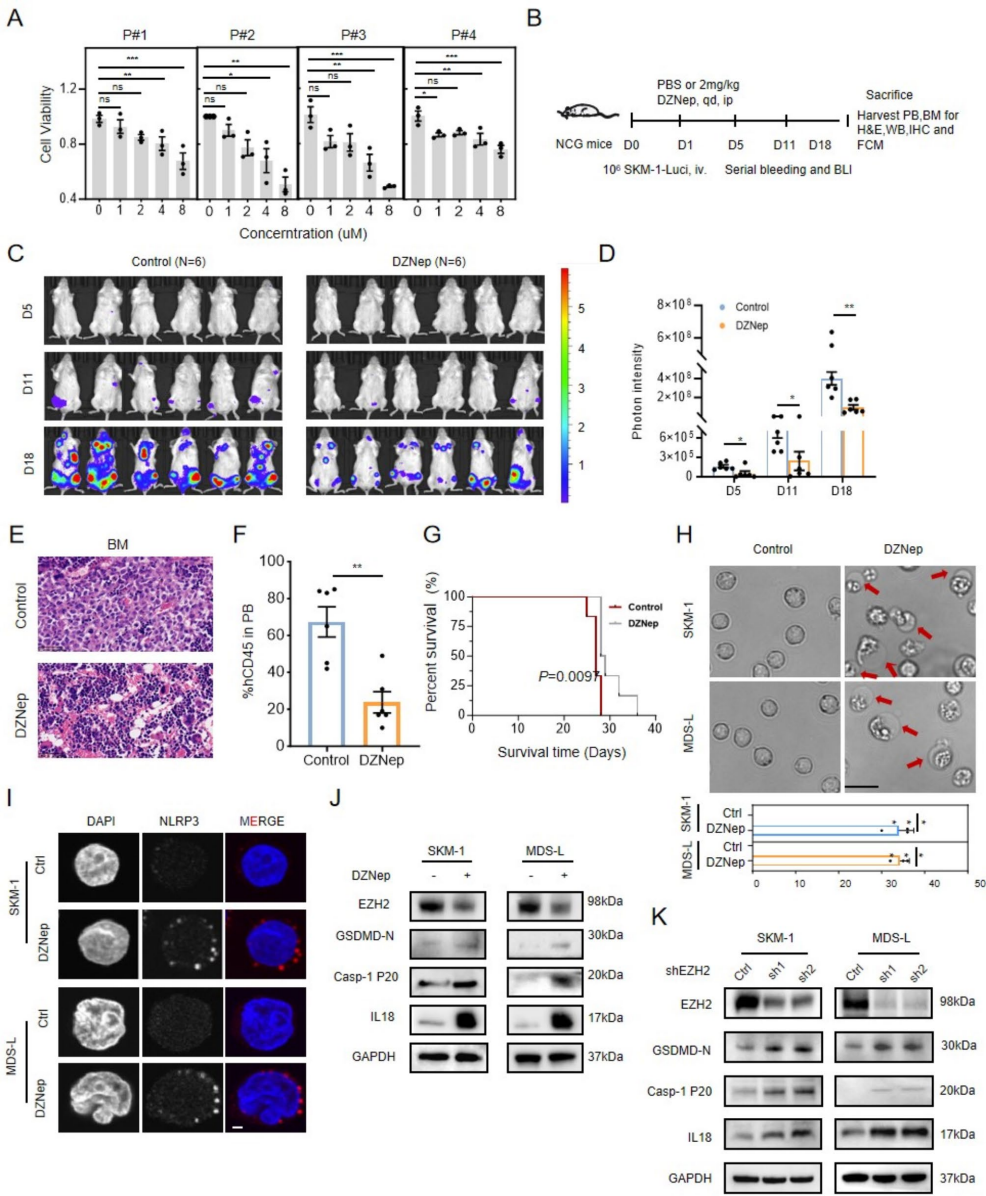
EZH2 inhibition induced MDS cells pyroptosis. **(A)** Cell viability of primary cells from higher-risk MDS patients (*n* = 4) treated with DZNep for 24 h. **(B)** Schematic diagram of in vivo treatment of SKM-1 transplanted mice. **(C-D)** Images of the MDS progression (C) and corresponding photon intensity of recipient mice **(D)**. **(E)** HE staining of the femur from recipient mice (40×). **(F)** The percentage of hCD45 cells in PB of recipient mice on day 18 (*n* = 6). **(G)** Survival analysis of SKM-1 transplanted mice treated with DZNep or vehicle. **(H)** Upper panel: the morphology of MDS cells treated with vehicle or 2µM DZNep for 48 h; the red arrows indicate the cells with bubbles. Lower panel: The proportion of cells with bubbles treated with or without DZNep in SKM-1 and MDS-L. Scale bar:20 μm. **(I)** The co-immunostaining against DAPI (blue) and NLRP3 (red) in MDS cells treated with 2µM DZNep for 48 h. Scale bar: 2 μm. **(J)** The cell lysates were harvested after treating 2µM DZNep in SKM-1 and MDS-L cells for 48 h and were blotted against pyroptosis related antibodies. Loading control is GAPDH. **(K)** The expression of the indicated proteins in SKM-1 and MDS-L cells after EZH2 knockdown via shRNA. Statistical analyses were performed using the unpaired Student’s t-test for two-group comparisons. We used ANOVA for comparison of more than two groups. Survival comparisons were performed using the Kaplan–Meier method and analyzed using the log-rank test. Each value represents mean ± SEM of three independent experiments. Ns, not significant, *P < 0.05, **P < 0.01, ***P < 0.001

By comparing the RNA expression profiles of cells with or without EZH2 knockdown, 58 differentially expressed genes were identified (Fig. 2A). Among them, RHA was reported to be involved in cell pyroptosis [6]. RHA levels decreased in EZH2-depleted cells while upregulation of RHA was found in EZH2-overexpressing cells (Fig. 2B-D). DZNep treatment showed a similar effect to EZH2 knockdown (Fig. 2E). Knockdown of RHA markedly suppressed MDS cell viability (Fig. S2A), and activated cell pyroptosis (Fig. S2B-C), suggesting RHA was involved in EZH2 inhibition induced cell pyroptosis.

**Fig. 2:**
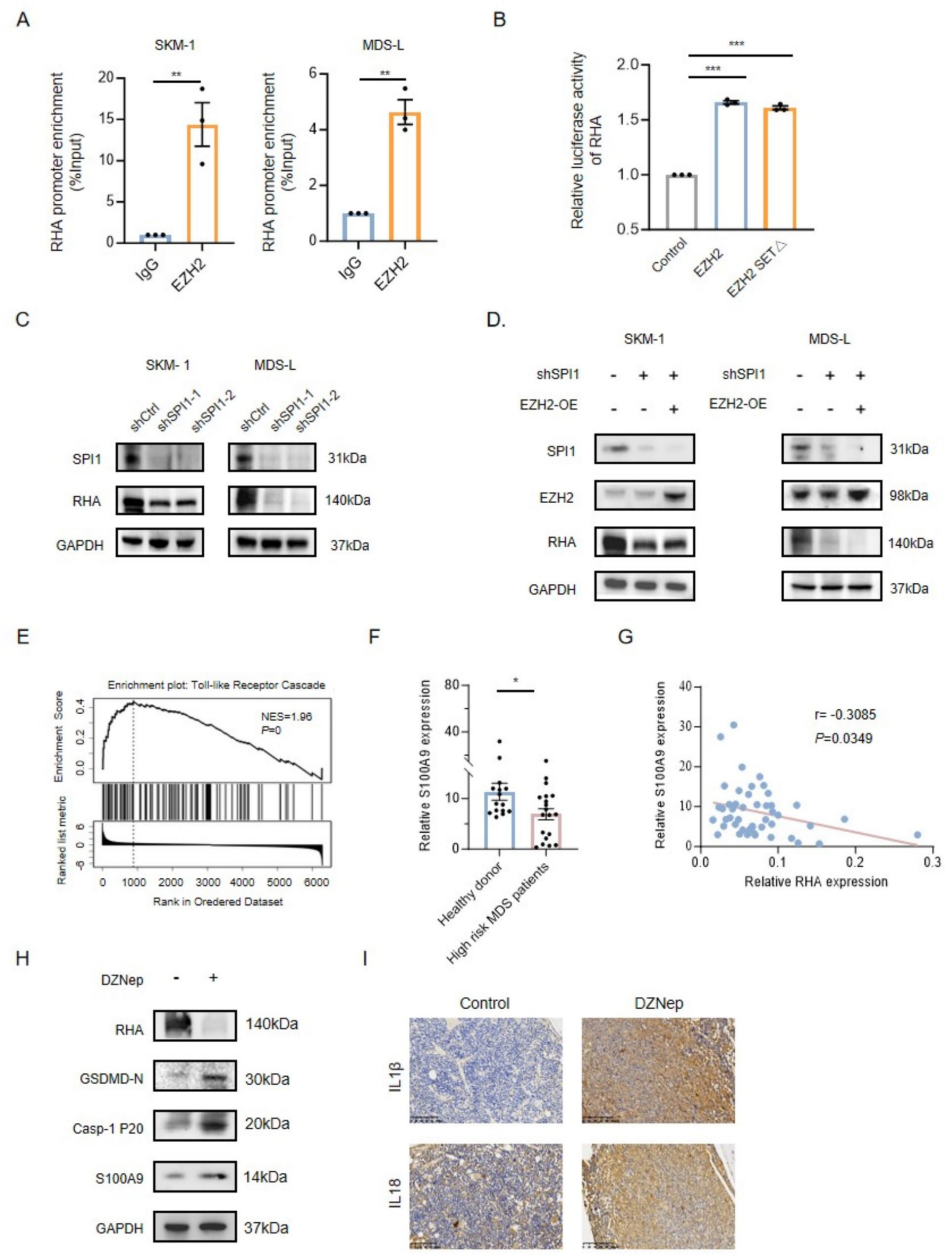
EZH2 Inhibition induced pyroptosis through RHA mediated S100A9 overexpression. **(A)** Venn diagram showed the overlapped downregulated genes after knocking down EZH2 (data from the GSE183458 and GSE112378 datasets, |log2FoldChange|≥1, *P* < 0.05). **(B-C)** The RHA mRNA **(B)** and protein expression **(C)** were downregulated in EZH2 depletion SKM-1 and MDS-L cells. **(D)** The RHA expression was upregulated in EZH2 overexpressed SKM-1 and MDS-L cells. **(E)** The RHA protein expression with or without DZNep in SKM-1 and MDS-L. **(F)** SPI1 was the only transcription factor (TF) that binds to both EZH2 protein and *RHA* promoter. Red circles represented EZH2 binding proteins found in MDS-L cells by IP in combination with MS. Blue circle was AnimalTFDB 4.0 predicted TFs that bind to the *RHA* promoter region. **(G)** EZH2 interacted with SPI1 in SKM-1 and MDS-L cells confirmed by Co-IP. **(H)** Enrichment of the *RHA* promoter by anti-SPI1 antibody in SKM-1 and MDS-L cells by ChIP-qPCR assay. **(I)** RHA luciferase reporter activity in 293T cells overexpressing EZH2 or SPI1. **(J)** Volcano plot of differentially expressed genes after RHA knockdown in MDS-L cells. The red dots represented upregulated differentially expressed genes (log2FC>1), the black dots represented downregulated differentially expressed genes (log2FC<-1), and the gray dots represented nonvariable genes (1< log2FC<-1). **(K)** The S100A9 protein expression in RHA depleted SKM-1 and MDS-L cells. **(L)** The co-immunostaining against DAPI (blue) and NLRP3 (red) in S100A9 overexpressed SKM-1 and MDS-L cells. Scale bar:20 μm. **(M)** Expression of pyroptosis-related protein in SKM-1 and MDS-L transduced with S100A9 overexpression vector or control vector. **(N)** The indicated protein expression after treatment of RHA-overexpressing cells with DZNep or vehicle. **(O)** Graphical abstract of EZH2 function in MDS. Statistical analyses were performed using the unpaired Student’s t-test for two-group comparisons and ANOVA for comparison of more than two groups. Each value represents mean ± SEM of three independent experiments. Ns, not significant, *P < 0.05, **P < 0.01, ***P < 0.001

GSK126 or Tazemetostat, both S-adenosyl-methionine competitive inhibitors, could inhibit the methyltransferase activity of EZH2 but without affecting EZH2 expression [7, 8]. GSK126 or Tazemetostat treatment didn’t impact RHA expression in MDS cells, suggesting EZH2 regulated RHA expression was independent of its HMT activity (Fig. S2D-E). We confirmed that EZH2 protein bound to *RHA* promoter DNA in MDS cells (Fig. S3A). Enforced expression of either wild-type EZH2 or an enzymatically inactive EZH2 SET domain deletion mutant activated RHA transcription (Fig. S3B). To gain insight into the mechanism of EZH2 regulating RHA expression, 23 EZH2 binding TFs were identified by co-IP coupled with mass spectrometry (MS) in MDS-L, among which, SPI1 was strongly predicted to bind to RHA (Fig. 2F). Indeed, SPI1 bound to the *RHA* promoter in MDS cells (Fig. 2G-H). Overexpression of either EZH2 or SPI1 activated RHA transcription, while coexpression of EZH2 and SPI1 showed the strongest RHA transcriptional activity (Fig. 2I). Importantly, SPI1 deletion inhibited RHA expression, which was not rescued by ectopic expression of EZH2 (Fig. S3C-D). These data indicated that EZH2 cooperated with SPI1 to induce RHA transcription.

To elucidate how RHA regulated pyroptosis, we compared the RNA profiles of MDS-L cells with or without RHA knockdown. Gene Set Enrichment Analysis (GSEA) showed significant positive enrichment of the Toll-like receptor cascades gene set, which associated with pyroptosis activation, in RHA-deleted MDS-L cells compared with the control cells (Fig. S3E). Among the top 20 differentially expressed genes, we focused on S100A9, which acted as a DAMP to initiate pyroptosis and a factor for inflammatory responses leading to defective erythropoiesis in MDS (Fig. 2J) [9, 10]. Accordingly, the S100A9 protein increased upon RHA knockdown in MDS cells (Fig. 2K). Enforced expression of S100A9 triggered pyroptosis of MDS cells as evidenced by increased NLRP3 level, and cleavage of GSDMD and caspase-1 proteins (Fig. 2L-M). The S100A9 mRNA expression was lower in BM cells from high-risk MDS patients than that from healthy donors and negatively connected with RHA expression (Fig. S3F-G). Overexpression of RHA significantly inhibited S100A9 expression, thereby suppressing pyroptosis induced by DZNep (Fig. 2N). More importantly, BM cells from DZNep treated mice transplanted with MDS cells showed increased S100A9, GSDMD-N, caspase-1 p20, IL18 and IL1β expression along with reduced RHA expression, which was consistent with in vitro results (Fig S3H-I). These results suggested that inhibition of EZH2 induced pyroptosis through RHA mediated S100A9 overexpression.

In summary, we found EZH2 promoted RHA expression together with SPI1, independent of its HMT activity. Decreased RHA expression induced by EZH2 inhibition resulted in S100A9 upregulation and subsequently initiated cell pyroptosis (Fig. 2O). Our results elucidated the EZH2 noncanonical function in gene expression and its role in cell pyroptosis of MDS cells. Suppressing the multifaceted activities of EZH2, such as the degradation of EZH2 by using EZH2-PROTAC [11], is a promising strategy against MDS.

## Electronic supplementary material

Below is the link to the electronic supplementary material.

Supplementary Material 1

Supplementary Material 2: Fig. 1. DZNep inhibits MDS cells proliferation in vitro. (A) Cell viability of MDS cell lines treated with DZNep (EZH2 inhibitor) for 24 h, 48 h, or 72 h. (B) Cell viability of SKM-1 and MDS-L cells treated with DZNep or various cell death inhibitors: SKM-1 and MDS-L cells were incubated with 1µM ferrostatin-1 (a ferroptosis inhibitor), 20µM ZVAD-FMK (an apoptosis inhibitor), 40µM VX765 (a pyroptosis inhibitor) or 20µM necrostatin-1 (a necroptosis inhibitor) for 1 h prior to DZNep for 72 h. Statistical analyses were performed using the ANOVA for comparison of more than two groups. Each value represents mean ± SEM of three independent experiments. Ns, not significant, *P < 0.05, **P < 0.01, ***P < 0.001. Fig. 2. EZH2 inhibition induced pyroptosis through downregulating RHA expression. (A)Cell viability in SKM-1 and MDS-L cells after RHA knockdown. (B) NLRP3 protein expression upon RHA knockdown in SKM-1 and MDS-L cells. Scale bar: 2 μm. (C) Immunoblots of the pyroptosis-related proteins in RHA knockdown SKM-1 and MDS-L cells. (D) RHA mRNA expression in SKM-1 and MDS-L treated with vehicle or 2 µM GSK126 (S-adenosyl-methionine competitive inhibitors) or TAZE (S-adenosyl-methionine competitive inhibitors) for 48 h. (E) RHA protein expression in SKM-1 and MDS-L treated with vehicle or 2 µM GSK126 and TAZE for 48 h. Statistical analyses were performed using the unpaired Student’s t-test for two-group comparisons. Each value represents mean ± SEM of three independent experiments. Ns, not significant, *P < 0.05, **P < 0.01, ***P < 0.001. Fig. 3. EZH2 and SPI1 upregulated RHA expression. (A) The enrichment of *RHA* promoter (TSS, designated − 1000 bp to -1500 bp) was found via ChIP-qPCR analysis in SKM-1 and MDS-L. (B) RHA luciferase reporter activity in 293T transfected with pGL3-RHA and EZH2 WT or SET△. (C) The RHA protein decreased in SPI1 knocked down SKM-1 and MDS-L cells. (D) RHA expression in SPI1-depleted cells followed by overexpression of EZH2. (E) GSEA for the TLR pathway gene signature in shRHA versus shctrl MDS-L cells. (F) The S100A9 mRNA level in BMNCs of MDS patients (*n* = 43) and healthy donors (*n* = 16). (G) The relevance between S100A9 and RHA expression in BMNCs of MDS patients via Pearson correlation test. (H) The indicated protein expression in BM cells derived from recipients treated with DZNep or vehicle. (I) IHC of femur from recipients treated with DZNep or vehicle using IL1β and IL18 antibody (20×). Statistical analyses were performed using the unpaired Student’s t-test for two-group comparisons and ANOVA for comparison of more than two groups. Each value represents mean ± SEM of three independent experiments. Ns, not significant, *P < 0.05, **P < 0.01, ***P < 0.001.

## Data Availability

No datasets were generated or analysed during the current study.

## Abbreviations

MDS: Myelodysplastic Syndromes
EZH2: enhancer of zeste homologue 2
RHA: RNA Helicase A
PB: Peripheral blood
AML: Acute myeloid leukemia
IPSS-R: Revised international prognostic scoring system
TF: Transcription factor
BMNCs: Bone marrow mononuclear cells
Co-IP: Co-immunoprecipitation
ChIP: Chromatin immunoprecipitation
DAMPs: Damage-associated molecular patterns
GSDMD: Gasdermin D
PROTAC: Proteolysis targeting chimera
