# Supplementary material for: EZH2 inhibition induces pyroptosis via RHA-mediated S100A9 overexpression in myelodysplastic syndromes
Source: Exp Hematol Oncol. 2025 Jan 29;14:9. doi: 10.1186/s40164-025-00600-3 (PMC11780917; doi:10.1186/s40164-025-00600-3)
Supplement: Supplementary file 1 — Supplementary Material 1 [file 40164_2025_600_MOESM1_ESM.docx]

**Supplemental Information to: EZH2 Inhibition Induces Pyroptosis via RHA-Mediated S100A9 Overexpression in Myelodysplastic Syndrome**

**Materials and methods**

Patient sample

Bone marrow mononuclear cells (BMNCs) specimens were gathered from *de novo* MDS patients diagnosed according to the 2016 World Health Organization (WHO) classification at the First Affiliated Hospital of Zhejiang University between February 2010 and May 2020^[13]^. Healthy donors were served as the control group. The primary patient information used in the cell viability experiments was arranged in Supplementary Table S1. This study was approved by the Ethics Committee of the First Affiliated Hospital of Zhejiang University (Ref. 2022-735).

Cell lines

The human MDS cell line MDS-L was kindly endowed by Dr. Kaoru Tohyama^[14]^ .The SKM-1 cell line was acquired from the Health Science Research Resources Bank (Osaka, Japan). Both cell lines were cultured in RPMI 1640 medium containing 10% fetal bovine serum (FBS) at 37℃ with 5% CO_2_, and the MDS-L cells were supplemented with 10ng/mL human recombinant IL-3.

Plasmid and transfection

The short hairpin RNA (shRNA) of EZH2 and RHA were synthesized into a psi-LVRU6GP vector (GeneCopoeia, MA,USA). The control, EZH2 and RHA sequences were listed in Supplementary Table S2. The coding DNA sequence of S100A9 (NM002965.4), RHA (NM001357.5) and EZH2 (NM004456.5) were cloned into pCDH-MSCV-MCS-EF1-GFP-T2A-Puro backbone (MiaoLing, Hubei, China). We also generated EZH2 SET domain deletion mutant (EZH2 SET△). Overexpression of SPI1 were constructed into CMV-MCS-EF1-GFP-Puro vector (TranSheepBio, Shanghai, China). 293T cells were added with compounds containing packaged plasmids to produce viral particles and collected at 48h and 72 h. SKM-1 and MDS-L cells were transfected at 72h and selected with puromycin.

Cell viability assay

CellTiter-Lumi™ Luminescent Cell Viability Assay Kit (Beyotime, Shanghai, China) was performed in accordance with the manufacturer’s instructions. Briefly, 100 μL cells were seeded into a black 96-well plate at a density of 1×10^5^ cells/mL, treated with drugs for different times, and then incubated at room temperature (RT) for 10min after shaking for 2min before detection.

Flow cytometric analysis

Harvest the peripheral blood (PB) of mice and wash with cold PBS, then incubated the human CD45 (hCD45) antibody (ab134199, Abcam, Cambridge, USA) in the dark at 4 °for 30 min after lysis of erythrocytes. HCD45 positive cells were tested by CytoFlex instrument (Beckman Coulter, CA, USA).

Real time quantitative polymerase chain reaction (qPCR)

After RNA was extracted by Trizol-based (Invitrogen, Carlsbad, USA) protocol. cDNA was done via a Reverse Transcription Kit (Takara, Tokyo, Japan). QPCR was conducted with SYBR Green qPCR Master Mix (Takara, Japan) employing CFX96 Real-Time PCR systems (Bio-Rad, Hercules, USA). The relative gene expression was measured by 2^-ΔΔCt^ method. All the primers were given in Supplementary Table S3.

Western Blot

The collected cells were lysed with radioimmunoprecipitation (RIPA) for 30min on ice and centrifuged at 4℃ for 15min. Part of supernatant was taken for quantification by BCA kit (Beyotime, China), the rest was boiled at 100℃ for 5 min with appropriate loading buffer (Invitrogen, USA). The mixture was subjected to SDS-PAGE gel, moved to PVDF membranes (Millipore, MA, USA) after electrophoresis, incubated with milk for 1h at RT before incubating primary antibody. Membranes were washed thrice with TBST followed by secondary antibody at RT and detected with ECL reagent (Thermo Fisher Scientific, MA, USA). All antibody information were provided in Supplementary Table S4.

Immunofluorescence

After washing the collected cells with PBS, the cells were fixed at RT with 4% paraformaldehyde for 20min. The cells were then dropped onto slides for air drying. Triton X-100 was soaked and blocked with 5% BSA for 1h, followed by incubation with primary antibodies. The slides were washed with PBS and incubated with the corresponding secondary antibodies. Finally, add DAPI (Beyotime, China) to the slides and cover it to avoid bubbles. Images were taken under an Olympus FV3000 confocal microscope. All antibody information were provided in Supplementary Table S4.

RNA-seq

Total RNA was isolated, purified with oligo (dT)-attached magnetic beads and synthesized into cDNA. The cDNA fragments were amplified by PCR, purified by Ampure XP Beads, and heated for denaturing to obtain the final library. The library products were sequenced on BGIseq500 platform (BGI-Shenzhen, China). Gene set enrichment analysis (GSEA) was conducted by WebGestalt.^[15]^

Co-immunoprecipitation (Co-IP)

Cells were lysed in RIPA buffer (Beyotime, China) followed by centrifugation at 13,000×g at 4℃ for 15min. The protein lysates were incubated with antibodies or rabbit IgG on a rorator overnight at 4℃. The magnetic beads (CST, Boston, USA) were washed three times and then added to the immune complex for additional 2h. Wash the complex three times and resuspended in 1×loading buffer (Invitrogen, USA) and boiled for 5min. The eluted proteins were analysed by western blot.

Co-IP and LC-MS analysis

Samples from immunoprecipitated with EZH2 antibodies from MDS-L cell lysates were electrophoretic on SDS-PAGE, stained with coomassie brilliant blue staining (Solarbio, Beijing, China). Cut the gel lanes and subjected to in-gel trypsin digestion followed by LC-MS with Q Exactive mass spectrometer (Thermo Fisher Scientific, USA). The data analyzed by Proteome Discoverer 2.1 (Thermo Scientific, MA, USA).

Chromatin immunoprecipitation (ChIP)-qPCR

ChIP was conducted using ChIP Assay Kit (Beyotime, China) according to the manufacturer’ instrucions. Anti SPI1 antibody or rabbit IgG (Beyotime, China) were utilized for immunoprecipitation. DNA-protein complexes were eluted, de-crosslinked, and heated for 4h at 65℃. DNA was purified with PCR purification kit (Beyotime, China) and subjected to qPCR assay. The primers for RHA promoter are as follows. RHA: (F) ATGTCTTCTAGGCCCCTGCT, (R) TGATCACTCCCTTTAAAACTGTCA^[16]^.

Luciferase reporter assay

The human *RHA* promoter was cloned from cDNA into pGL3-basic (Promega, Madison, USA). 293T cells were seed in a 48-well plate and cotransfected with 125ng pGL3-RHA, 125ng pCDH-EZH2 or CMV-SPI1 or EZH2 SET△ and 12.5ng pRL-TK using Neofect DNA transfection reagent (Beijing, China). Luciferase activities were measured 48h post-transfection by Dual-Luciferase Assay System (Promega, USA). The firefly luciferase activity was normalized to the Renilla luciferase activity.

Mice model

Female immunodeficient NOD-Prkdc^em26Cd52^IL2rg^em26Cd22^/Nju (NCG) mice were purchased from GemPharmatech Co, Ltd (Nanjing, China) and bred in Hangzhou Medical College Laboratory Animal Center. Twelve mice were randomly divided into two groups, each injected with 1 million SKM-1-Luciferase (SKM-1-Luc) cells. The experimental group received intraperitoneal 2mg/kg DZNep (MCE, Shanghai, China) and the control group received the same volume of PBS daily. Tumor burden was monitored by IVIS Lumina LT system (PerkinElmer, California, USA) after injecting 150mg/kg D-luciferin (Promega, USA). The BM cells were obtained after the mice were euthanized with paralysis of the lower limbs.

Statistical analysis

GraphPad Prism 8.0 and SPSS 20.0 were applied for statistical analysis. Chi-square, Fisher's exact test or nonparametric test was used to analyze the relationship between gene expression level and clinical information. The Cox proportional hazards model was performed to assess the independent prognostic factors. Survival analysis was carried out by log-rank test. All experiments were repeated in triplicate, data were represented as mean ± SEM. Student *t*-test or multiple-group ANOVA was adopted for statistical analyses. *P* < 0.05 were considered statistically significant. * *P* < 0.05; ** *P* < 0.01; *** *P* < 0.001.

**Supplementary Tables**

**Table S1.** **Clinical characteristics of primary MDS patients**

| No. | Gender | Age | BM Blast | IPSS-R | Mutation | Karyotype |
| --- | --- | --- | --- | --- | --- | --- |
| #1 | Male | 62 | 13% | High risk | *BCOR, IDH1*, *PHF6*, *RUNX1* | 47, XY, +8 |
| #2 | Male | 67 | 7% | High risk | *ASXL1*, *PHF6*, *STAG2*, *TET2*, *ZRSR2* | 46, XY |
| #3 | Male | 76 | 12% | Very high risk | *IDH1*, *SRSF2*, *TP53* | -X, -Y, +2, del(2)(p11.2),+4,del(4)(p14),-5,+7,der(7;11)(p20;q10),+8,add(9)(p13),+11,+13,+14,-16,add(16)(p13.1),-18,add(19)(p13),+20,-21 |
| #4 | Male | 66 | 8.6% | High risk | *BCOR*, *RUNX1*, *U2AF1* | 48, XY, +8*2[2]/46, XY |

**Table S2. ShRNA sequences**

| Gene name | Sequence (5’-3’) |
| --- | --- |
| EZH2 sh1 | GCAAGAACTGCAGTATTCAGC |
| EZH2 sh2 | GCATCTATTGCTGGCACCATC |
| RHA sh1 | GCTGAAACAAGCATTACCATA |
| RHA sh2 | CCATCCTATGAAATTAGAGCA |
| SPI1 sh1 | AGAGCTTCGCCGAGAACAACT |
| SPI1 sh2 | CGGATCTATACCAACGCCAAA |

**Table S3. Primer sequences for qPCR**

| Gene | Forward Primer (5'-3') | Reverse Primer (5'-3') |
| --- | --- | --- |
| EZH2 | CGCTTTTCTGTAGGCGATGT | TGGGTGTTGCATGAAAAGAA |
| RHA | CGAACCATCTCAGCGACAAAA | TGAGGTCCATGCTTATTTGCTC |
| S100A9 | ATCATCAACACCTTCCACCAA | AGGTCCTCCATGATGTGTTCT |
| GAPDH | GGAGCGAGATCCCTCCAAAAT | GGCTGTTGTCATACTTCTCATGG |

**Table S4. Information for reagents**

| Reagent | Source | Identifier |
| --- | --- | --- |
| DZNep | MedChemExpress | HY-12186 |
| GSK126 | MedChemExpress | [HY-13470](https://www.medchemexpress.cn/GSK126.html) |
| Ferrostatin-1 | MedChemExpress | HY-100579 |
| Necrostatin-1 | MedChemExpress | HY-15760 |
| VX765 | MedChemExpress | HY-13205 |
| Tazemetostat | Selleck | S7128 |
| EZH2 | CST | #5246 |
| H3 | CST | #4499 |
| H3K27me3 | CST | #9733 |
| RHA | proteintech | 17721-1-AP |
| S100A9 | proteintech | 26992-1-AP |
| NLRP3 | proteintech | 19771-1-AP |
| GAPDH | proteintech | HRP-60004 |
| Caspase-1 | ABCAM | #4199 |
| GSDMD | ABCAM | #36425 |
| IL18 | ABCAM | Ab1842 |
| Human CD45 antibody | ABCAM | ab134199 |
| SPI1 | ABCAM | ab302623 |
| Reverse Transcription Kit | TaKaRa | RR036A-1 |
| SYBR Green qPCR Master Mix | TaKaRa | RR420 |
| DAPI | Beyotime | P0131 |
