# Supplementary material for: EZH2 inhibition induces pyroptosis via RHA-mediated S100A9 overexpression in myelodysplastic syndromes
Source: Exp Hematol Oncol. 2025 Jan 29;14:9. doi: 10.1186/s40164-025-00600-3 (PMC11780917; doi:10.1186/s40164-025-00600-3)
Supplement: Supplementary file 2 — Supplementary Material 2: Fig. 1. DZNep inhibits MDS cells proliferation in vitro. (A) Cell viability of MDS cell lines treated with DZNep (EZH2 inhibitor) for 24 h, 48 h, or 72 h. (B) Cell viability of SKM-1 and MDS-L cells treated with DZNep or various cell death inhibitors: SKM-1 and MDS-L cells were incubated with 1µM ferrostatin-1 (a ferroptosis inhibitor), 20µM ZVAD-FMK (an apoptosis inhibitor), 40µM VX765 (a pyroptosis inhibitor) or 20µM necrostatin-1 (a necroptosis inhibitor) for 1 h prior to DZNep for 72 h. Statistical analyses were performed using the ANOVA for comparison of more than two groups. Each value represents mean ± SEM of three independent experiments. Ns, not significant, *P < 0.05, **P < 0.01, ***P < 0.001. Fig. 2. EZH2 inhibition induced pyroptosis through downregulating RHA expression. (A)Cell viability in SKM-1 and MDS-L cells after RHA knockdown. (B) NLRP3 protein expression upon RHA knockdown in SKM-1 and MDS-L cells. Scale bar: 2 μm. (C) Immunoblots of the pyroptosis-related proteins in RHA knockdown SKM-1 and MDS-L cells. (D) RHA mRNA expression in SKM-1 and MDS-L treated with vehicle or 2 µM GSK126 (S-adenosyl-methionine competitive inhibitors) or TAZE (S-adenosyl-methionine competitive inhibitors) for 48 h. (E) RHA protein expression in SKM-1 and MDS-L treated with vehicle or 2 µM GSK126 and TAZE for 48 h. Statistical analyses were performed using the unpaired Student’s t-test for two-group comparisons. Each value represents mean ± SEM of three independent experiments. Ns, not significant, *P < 0.05, **P < 0.01, ***P < 0.001. Fig. 3. EZH2 and SPI1 upregulated RHA expression. (A) The enrichment of RHA promoter (TSS, designated − 1000 bp to -1500 bp) was found via ChIP-qPCR analysis in SKM-1 and MDS-L. (B) RHA luciferase reporter activity in 293T transfected with pGL3-RHA and EZH2 WT or SET△. (C) The RHA protein decreased in SPI1 knocked down SKM-1 and MDS-L cells. (D) RHA expression in SPI1-depleted cells followed by overexpression of E [file 40164_2025_600_MOESM2_ESM.docx]

**Supplemental Information to: EZH2 Inhibition Induces Pyroptosis via RHA-Mediated S100A9 Overexpression in Myelodysplastic Syndrome**

**Fig. S1**

**
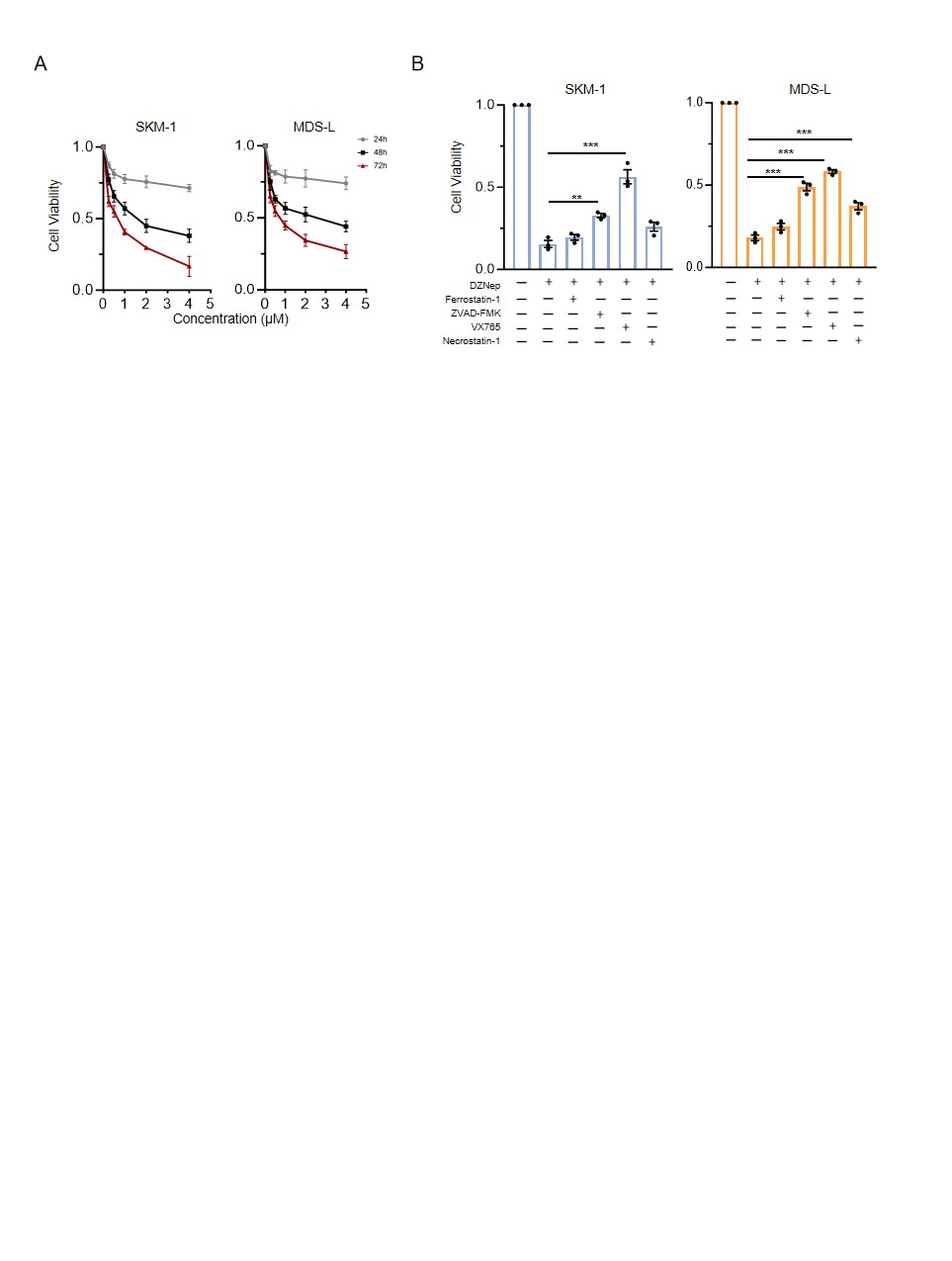
**

**Fig. 2**

**
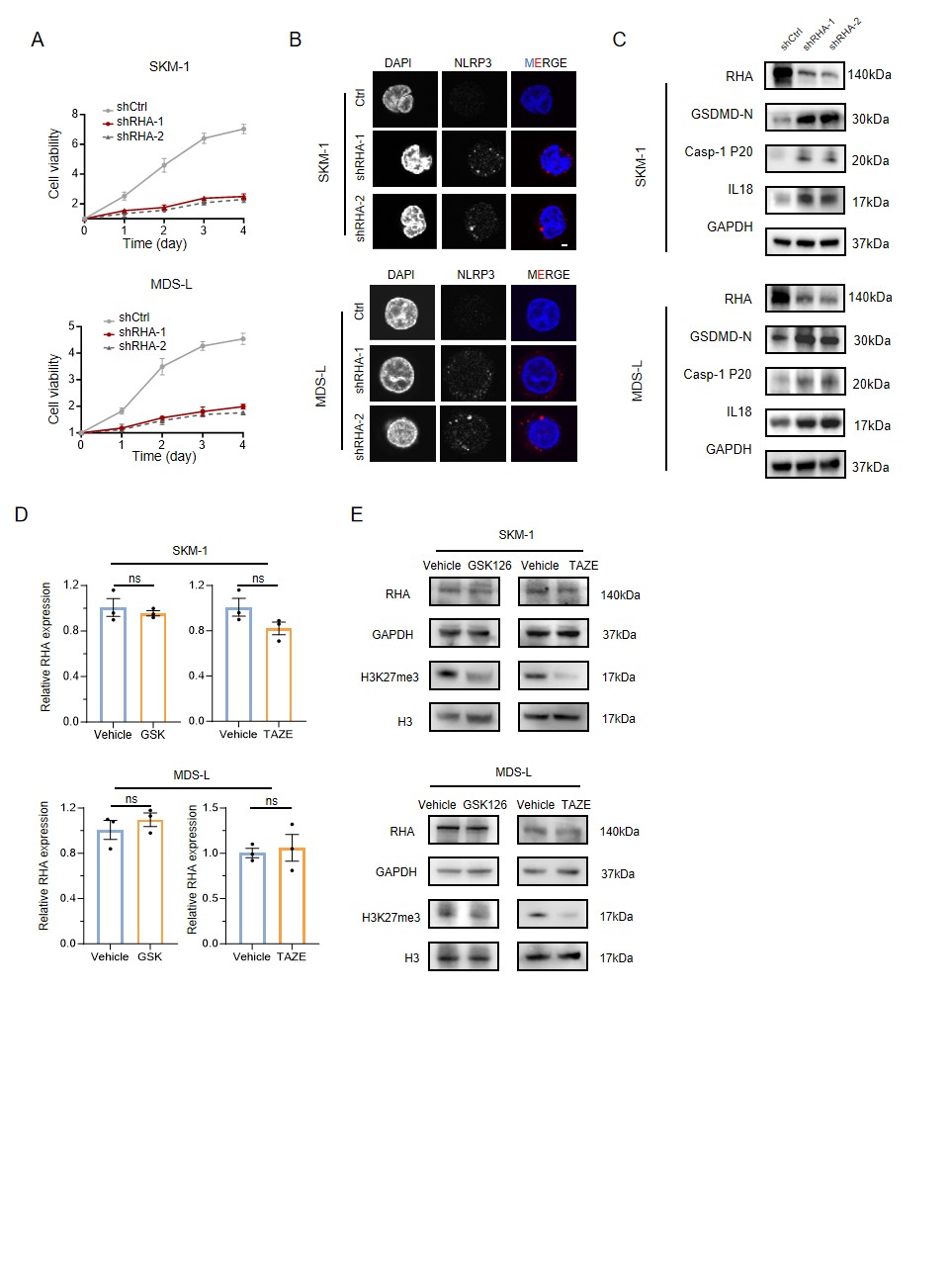
**

**Fig. 3**

**
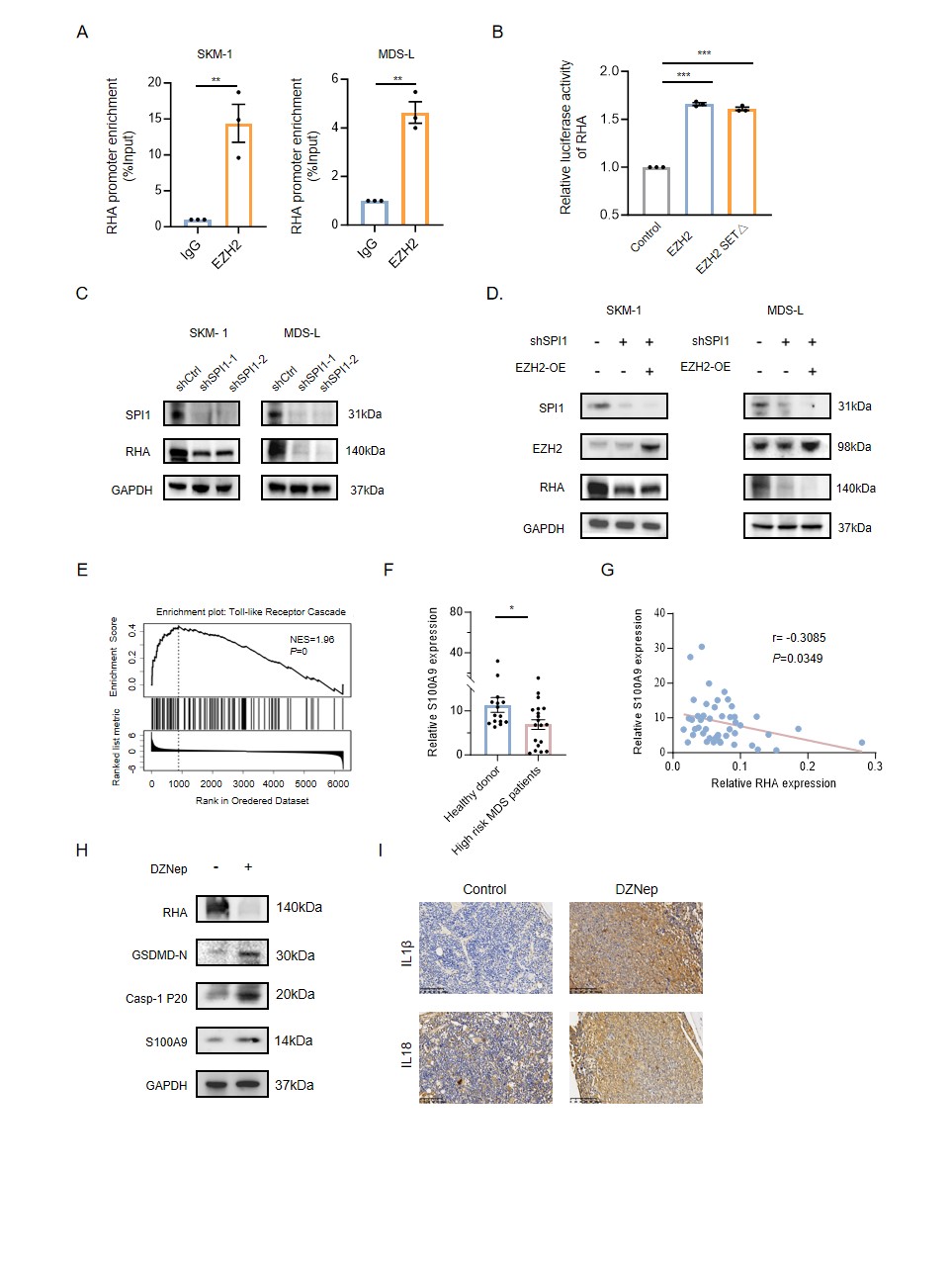
**
